# Increased functional connectivity in intrinsic neural networks in individuals with aniridia

**DOI:** 10.3389/fnhum.2014.01013

**Published:** 2014-12-19

**Authors:** Jordan E. Pierce, Cynthia E. Krafft, Amanda L. Rodrigue, Anastasia M. Bobilev, James D. Lauderdale, Jennifer E. McDowell

**Affiliations:** ^1^Department of Psychology, University of GeorgiaAthens, GA, USA; ^2^Department of Neuroscience, University of GeorgiaAthens, GA, USA; ^3^Department of Cellular Biology, University of GeorgiaAthens, GA, USA

**Keywords:** functional connectivity, aniridia, PAX6, resting state, dual regression, fMRI

## Abstract

Mutations affecting the PAX6 gene result in aniridia, a condition characterized by the lack of an iris and other panocular defects. Among humans with aniridia, structural abnormalities also have been reported within the brain. The current study examined the functional implications of these deficits through “resting state” or task-free functional magnetic resonance imaging (fMRI) in 12 individuals with aniridia and 12 healthy age- and gender-matched controls. Using independent components analysis (ICA) and dual regression, individual patterns of functional connectivity associated with three intrinsic connectivity networks (ICNs; executive control, primary visual, and default mode) were compared across groups. In all three analyses, the aniridia group exhibited regions of greater connectivity correlated with the network, while the controls did not show any such regions. These differences suggest that individuals with aniridia recruit additional neural regions to supplement function in critical intrinsic networks, possibly due to inherent structural or sensory abnormalities related to the disorder.

## Introduction

Aniridia is a panocular disorder (for a review see Lee et al., 2008; Hingorani et al., 2012), which is characterized by the absence or hypoplasia of the iris for which it is named, lens opacification, increased intraocular pressure, corneal keratopathy, foveal hypoplasia, and optic nerve hypoplasia (Nelson et al., 1984; McCulley et al., 2005). Symptoms are present in early childhood and worsen throughout the lifespan, with many individuals eventually developing cataracts and/or glaucoma (Hingorani et al., 2009, 2012; Gramer et al., 2012). In humans the development of aniridia is linked to PAX6, a highly conserved paired-box transcription factor that controls key steps in eye development in metazoans (Halder et al., 1995; Gehring, 1996; van Heyningen and Williamson, 2002). Heterozygous null mutations within PAX6, cytogenetic deletions of chromosome 11p13 that encompass PAX6 or chromosomal rearrangements that prevent PAX6 expression from one allele are all causal for aniridia (Ton et al., 1991; Fantes et al., 1995; Lauderdale et al., 2000; van Heyningen and Williamson, 2002; Robinson et al., 2008).

In addition to its well-known function in eye development, PAX6 plays several roles in the developing brain and spinal cord (Walther and Gruss, 1991; Osumi et al., 2008), including control of proliferation, specification, and differentiation of neural progenitor cells (Götz et al., 1998; Warren et al., 1999; Estivill-Torrus et al., 2002; Heins et al., 2002). In mice, mutations in PAX6 are associated with developmental abnormalities of the brain (Boretius et al., 2009; Tuoc et al., 2009; Georgala et al., 2011). In humans, abnormalities in brain structure have been reported for individuals with aniridia based on analyses of structural magnetic resonance images including: reduced volume of the interhemispheric commissures and corpus callosum (Sisodiya et al., 2001; Free et al., 2003; Bamiou et al., 2007), absence or hypoplasia of the pineal gland and olfactory bulb, polymicrogyria (Mitchell et al., 2003), reduced overall gray matter volume (Sisodiya et al., 2001), and reduced gray matter concentration in the cerebellum and the occipital lobes (Free et al., 2003).

In addition to structural abnormalities, basic functional deficits in vision (as described above (Lee et al., 2008)), audition (impaired interhemispheric transfer of auditory signals (Bamiou et al., 2004, 2007)), and olfaction (mild hyposmia to anosmia (Sisodiya et al., 2001)) have been reported previously. Few studies, however, have examined higher cognitive processes in this population. General cognitive functioning appears to be preserved in aniridia (Thompson et al., 2004) except in cases of Wilms tumor-aniridia-genitourinary-mental retardation (WAGR) syndrome (Schwartz et al., 1994; Fischbach et al., 2005); however, using measures of verbal executive function and social cognition, Heyman et al. (1999) reported impaired cognitive performance in a family with aniridia compared to healthy controls. These same individuals later participated in the only previous functional magnetic resonance imaging (fMRI) study published to date on humans with aniridia (Ellison-Wright et al., 2004). Seven affected family members were tested on verbal fluency and response inhibition tasks. The subjects with aniridia exhibited less activation of fronto-striatal-thalamic circuitry than controls during the task. Additionally, structural abnormalities including excess gray matter and reduced white matter were observed that suggested a potential link between the underlying neural architecture, cognitive performance, and patterns of functional activation.

The sensory deficits associated with the population of interest present challenges in using traditional fMRI stimuli to evaluate cognitive and sensory processing. One means of overcoming this obstacle is to implement task-free imaging techniques to measure intrinsic neural activation patterns while the participant is in a relaxed, awake condition commonly known as “resting-state” (Beckmann et al., 2005; Fox et al., 2005; Damoiseaux et al., 2006). When a participant has no specific task to complete, thoughts are free to wander and cognition may periodically become focused within certain intrinsic domains. Accordingly, widespread neural networks that regularly function together during active behaviors will spontaneously co-activate at low frequencies during a task-free fMRI session. These patterns of activity can be measured via the blood oxygenation level dependent (BOLD) signal, and inter-correlated to determine the intrinsic functional connectivity of various regions across the cortex (Biswal et al., 1995; Greicius et al., 2003; Damoiseaux et al., 2006; De Luca et al., 2006; Fox and Raichle, 2007; Rogers et al., 2007). Furthermore, this inherent neural synchrony has been related to activity during cognitive tasks (Seeley et al., 2007; Sharp et al., 2011; Kannurpatti et al., 2012; Sala-Llonch et al., 2012) and, thus, can provide insight into cognitive function without the need for stimulus presentation, task compliance and/or overt behavioral measures.

In the present study three well-documented intrinsic connectivity networks (ICNs; Seeley et al., 2007) were investigated: the executive control network, the primary visual network, and the default mode network. The executive control network (Greicius et al., 2003; Beckmann et al., 2005; Damoiseaux et al., 2006; Seeley et al., 2007), based in frontal cortex, relates to cognitive control processes and mediates activity in other brain regions; it was chosen due to previously reported differences in verbal executive control and frontal activation associated with aniridia (Ellison-Wright et al., 2004). The primary visual ICN includes neurons that process visual sensory input in its early stages and has been shown to be disrupted in individuals with visual deficits (Liu et al., 2007; Yu et al., 2008; Qin et al., 2013). Finally, the default mode network (Raichle et al., 2001) represents ongoing neural processes including self-referential thought (Gusnard et al., 2001; Fransson, 2005; Kim, 2010) and memory retrieval (Greicius et al., 2003; Buckner et al., 2008), and is often found to be anti-correlated with brain activity during demanding cognitive tasks (Fox et al., 2005). It was hypothesized that the functional connectivity for the aniridia group would be reduced in all ICNs relative to the comparison group due to their altered sensory inputs, neural development, and structural organization. Characterizing the synchrony of neural networks during task-free fMRI in individuals with aniridia, in conjunction with previously identified structural abnormalities in this population, can elucidate differences in neural function that result from the disruption of the PAX6 protein in this condition.

## Materials and methods

### Participants and procedure

Fourteen individuals with aniridia and 15 healthy controls participated in the current study. Data from two participants with aniridia were excluded from analyses (due to a significant dropout artifact and missing data). One control subject was excluded due to an anatomical abnormality, and two others were excluded because they did not match the demographic profile of an individual in the aniridia group. This left 12 individuals with aniridia (7 females, mean age = 36 years, SD = 15) and 12 age- and gender-matched controls (7 females, mean age = 35 years, SD = 14) who were included in the analyses. Controls were recruited through flyers posted in the community. Participants with aniridia were recruited through the Aniridia Foundation International Conference held in 2011 and were clinically diagnosed with aniridia. Three of the participants with aniridia belonged to the same family; all other participants included in the analyses were unrelated. After written informed consent was obtained, 10 mL blood samples were collected from each participant with aniridia for genotyping. All participants then completed an MRI session where a high-resolution structural image and a task-free functional MRI scan were collected. During the functional scan, participants were instructed simply to keep their eyes closed without falling asleep. All activities were approved by the Institutional Review Board of the University of Georgia.

### Genotyping

Buffy coat was extracted from each blood sample, and genomic DNA (gDNA) was isolated and aliquoted in EDTA storage tubes. Each participant’s gDNA was amplified and all coding and noncoding exons of the PAX6 gene (exons 1 through 13) and the alternatively spliced exon 5a were directly sequenced and analyzed. Sequence was compared to the wild-type PAX6 cDNA sequence and mutations were identified and are reported in the nomenclature system recommended by den Dunnen and Antonarakis (2001). Eight of the 12 participants with aniridia in the current study have confirmed heterozygous loss-of-function mutations within the coding region of the PAX6 gene. The remaining four individuals had chromosomal rearrangements that are expected to abolish PAX6 expression from the variant allele, but this has not been directly tested. Clinically, the eye phenotypes of these four individuals were indistinguishable from those with confirmed nonsense mutations. The demographic and genotypic information of each participant with aniridia is provided in Table 1.

**Table 1:** **Demographic information on all 12 participants with aniridia**.

Subject	Age	Gender	Handedness	Confirmed mutation in PAX6
1	18	Male	Right	No
2	19	Female	Ambidextrous	Yes (c.771delG)
3*	20	Male	Right	Yes (c.204delC)
4*	24	Female	Left	Yes (c.204delC)
5	25	Female	Left	No
6	28	Female	Right	Yes (c.28C>T)
7	39	Male	Right	Yes (c.481delG)
8	47	Male	Right	No
9*	47	Male	Left	Yes (c.204delC)
10	51	Female	Right	Yes (c.766-3C>G)
11	53	Female	Right	No
12	60	Female	Ambidextrous	Yes (c.799A>T)

*Indicates individuals from a single family.

### Imaging parameters

All data were collected on a 3T GE Signa MRI (General Electric, Milwaukee, WI, USA) at the University of Georgia’s Bio-Imaging Research Center. For the high resolution structural scan, images were acquired with a T1-weighted 3D FSPGR sequence (echo time (TE) = min full, flip angle = 20°, field of view (FOV) = 240 mm × 240 mm, matrix size = 256 × 256, 150 axial slices, in-slice resolution = 0.94 × 0.94 mm, slice thickness = 1.2 mm). For the task-free functional scan, a T2*-weighted gradient echo whole-brain EPI sequence was acquired (repetition time (TR) = 5000 ms, TE = 25 ms, flip angle = 90°, FOV = 256 mm × 256 mm, matrix size = 128 × 128, 55 oblique slices, in-slice resolution = 2 × 2 mm, slice thickness = 2.4 mm, 105 volumes, total scan time = 8 min 45 s). A TR of 5000 ms was determined to be necessary to collect 55 slices per volume with whole-brain coverage, while being short enough to capture the low-frequency fluctuations of the signal of interest. The first four volumes were discarded to allow for scanner stabilization. The functional scan was collected with oblique slices aligned to a plane containing the superior margin of the anterior commissure and inferior margin of the posterior commissure for each participant.

### Data analysis

Functional MRI analyses were conducted using a procedure previously implemented in our laboratory (Krafft et al., 2014) utilizing FMRIB Software Libraries (FSL, version 5.0.1; Oxford, United Kingdom) and Analysis of Functional NeuroImages (AFNI; Cox, 1996) software packages. The following preprocessing was applied in FSL for each participant: motion correction using MCFLIRT (Jenkinson et al., 2002), non-brain removal using BET (Smith, 2002), spatial smoothing (full-width at half-maximum [FWHM] = 4 mm), grand-mean intensity normalization, and high-pass temporal filtering (0.01 Hz). Registration was carried out using FLIRT (Jenkinson and Smith, 2001; Jenkinson et al., 2002), with which each functional run was aligned to the individual’s structural image, transformed into MNI152 standardized space, and resampled to 2 mm isotropic voxels. Probabilistic independent components analysis (ICA; Beckmann and Smith, 2004) was conducted in FSL’s MELODIC for each participant to denoise individual data. Components that represented noise were selected by spatial and temporal characteristics as detailed by Kelly et al. (2010) (including head motion, physiological noise, or scanner artifacts) and removed.

Between-subjects analysis was carried out using a dual regression approach that allows for voxel-wise comparisons of intrinsic functional connectivity (Beckmann et al., 2009; Filippini et al., 2009; Zuo et al., 2010). Briefly, this approach first identifies patterns of brain activity common to all participants and subsequently finds the activity on the individual level associated with that common network. This then allows for group comparisons to be made throughout the whole brain on the strength and extent of functional correlation with the main network. To prepare the data for the dual regression, individuals’ preprocessed data were temporally concatenated across subjects to create a single 4D (three spatial dimensions × time) dataset. The concatenated dataset was decomposed using group ICA to identify large-scale patterns of functional synchrony in the entire sample of 24 participants. Forty spatially-independent components were identified using automatic dimensionality estimation. Components of interest were selected by identifying the component with the greatest spatial correlation (using FSL’s “fslcc” command) with previously identified ICNs from the publicly available dataset of Beckmann et al. (2005) and visually confirmed (e.g., Smith et al., 2009; Kiviniemi et al., 2011; Ray et al., 2013).

Dual regression in FSL then was used to identify, within each subject’s fMRI dataset, subject-specific temporal dynamics and associated spatial maps for each group component. This technique involved two steps. First, the full set of 40 group-ICA spatial maps (derived from both aniridia and control participants’ data) was used in a linear model fit (spatial regression) against each individual’s “denoised” fMRI dataset. This resulted in a matrix for each participant describing that individual’s temporal dynamics for each ICA component. In the second step, each of the individuals’ time courses from the first step was normalized to unit variance and used in a linear model fit (temporal regression) against their own fMRI dataset to estimate the participant’s spatial maps for each component. These subject-specific whole-brain spatial maps reflected the degree to which each voxel was functionally correlated with the group-level network, and were transformed into *Z*-maps for group comparison. A two-sample *t*-test was then performed between the aniridia and control groups on the individual *Z-maps* corresponding to the three group components of interest. The statistical maps from these *t*-tests were thresholded at a voxel-wise uncorrected *p* < 0.005. To protect against false positives, a thresholding method in AFNI derived from Monte Carlo simulations (accounting for the smoothness of the data) then was applied to the statistical maps (Ward, 2000). Based on these simulations, a family-wise alpha of 0.05 was preserved with three-dimensional clusters with a minimum volume of 55 voxels.

## Results

**Figure 1:**
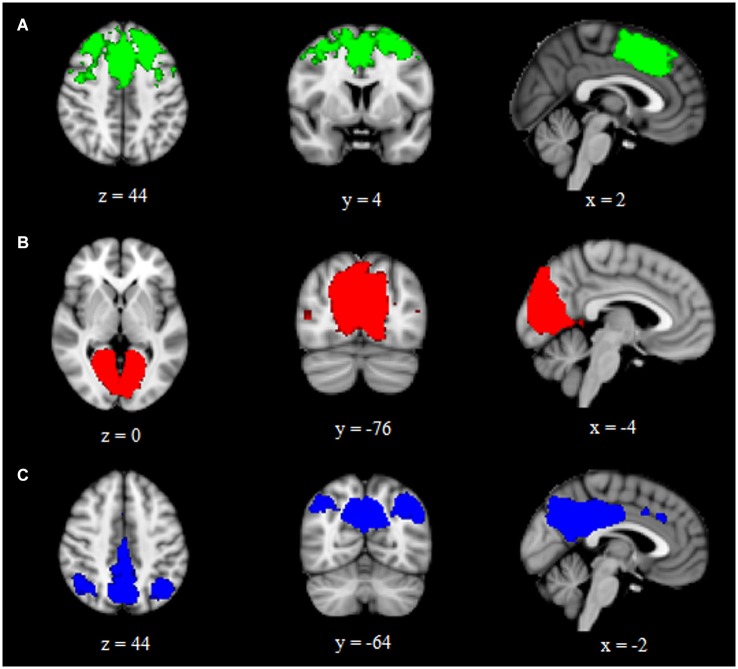
**Group ICA components representing (A) executive control, (B) primary visual, and (C) default mode intrinsic connectivity networks**. Images are overlaid on the MNI152 brain and are presented in radiological orientation (right is left).

Using ICA and dual regression, this study examined differences in intrinsic functional connectivity networks between individuals with aniridia and healthy controls. Groups did not differ in the amount of head motion during the scans: aniridia participants moved an average of 0.52 mm (SD = 0.31) and control participants moved 0.67 mm (SD = 0.76), *t*_(22)_ = −0.61, *p* > 0.05. The two groups also did not differ in the percent of components removed as “noise” during individual ICA (aniridia = 49% (SD = 13); control = 41% (SD = 11), *t*_(22)_ = 1.60, *p* > 0.05). Of the 40 ICA components identified in the group analysis of all 24 participants, three were selected that represented networks of interest based on visual inspection and spatial correlation with previously identified networks (Beckmann et al., 2005). These three components corresponded to the executive control network, the primary visual network, and the default mode network (Figure 1). These components correlated with Beckmann et al. (2005) ICNs with Pearson correlation values of 0.50, 0.76, and 0.64, respectively.

**Figure 2:**
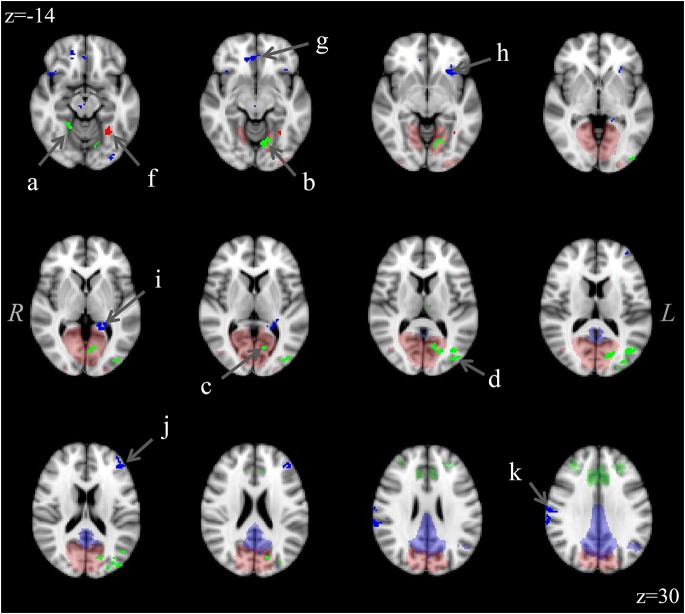
**Differences in functional connectivity within the three ICNs (executive control: green; visual: red; default mode: blue) showing greater connectivity in the aniridia group compared to controls**. Difference clusters are shown in bright, opaque hues in the foreground while group ICNs are shown in transparent hues on the anatomical background image. The executive control network clusters were located in: (a) right culmen/dentate, (b) left lingual gyrus, (c) left cuneus, (d) left middle occipital gyrus; and (e) right precuneus (not shown); the visual network cluster was located in (f) left fusiform gyrus; and the default mode network clusters were located in: (g) right medial frontal gyrus, (h) left inferior frontal gyrus, (i) left parahippocampal gyrus, (j) left middle frontal gyrus; and (k) right postcentral gyrus. T-maps were thresholded to a voxel-wise *p* < 0.005 and clustered to ensure a family-wise *p* < 0.05. The control group showed no regions of greater connectivity. Images are overlaid on the MNI152 brain and are presented in radiological orientation (right is left).

The analysis for each of the three ICNs showed the same pattern: participants with aniridia exhibited regions of greater functional connectivity than control participants, with no instances of controls showing greater connectivity than the aniridia group. The between group *t*-tests on the executive control network revealed multiple regions where the aniridia participants had greater functional connectivity with the network: the left middle occipital gyrus, left lingual gyrus, right culmen/dentate of the cerebellum, and right precuneus. For the primary visual network, the aniridia group showed greater connectivity in left fusiform gyrus, and for the default mode network the aniridia group had greater connectivity with right postcentral gyrus, left parahippocampal gyrus, right medial frontal gyrus, left middle frontal gyrus and left inferior frontal gyrus (Figure 2). Table 2 shows the location and size of the regions that differed between the groups for all three networks, with each cluster surpassing the *p* < 0.05 family-wise error rate threshold.

**Table 2:** **Location of significant connectivity differences**.

Intrinsic connectivity network	Anatomical location of cluster	Peak *t*-statistic MNI coordinates (*x,y,z*)	Size	(voxels)
Executive control	Left middle occipital gyrus	−40, −86, 2	223
	Left cuneus	−10, −70, 2	124
	Right culmen/dentate	18, −52, −32	101
	Left lingual gyrus	−10, −70,−10	81
	Right precuneus	20, −8, 48	71
Primary visual	Left fusiform gyrus	−28, −52, −16	56
Default mode	Right postcentral gyrus	68, −34, 26	154
	Left parahippocampal gyrus	−18, −44, 4	77
	Right medial frontal	18, 38, −18	74
	Left middle frontal gyrus	−40, 50, 16	71
	Left inferior frontal gyrus	−32, 20, −6	64

*MNI coordinates of the peak t-statistic and the voxel count of each cluster where the aniridia group showed greater connectivity than the control group with each of the three intrinsic connectivity networks of interest. Voxels were 2 mm^3^ in size and clusters had to contain at least 55 voxels to meet the criteria for significance using a family-wise error rate of p < 0.05*.

## Discussion

This study investigated intrinsic functional connectivity during task-free fMRI in individuals with aniridia compared to healthy age- and gender-matched controls. All participants in the aniridia group had a confirmed clinical diagnosis of aniridia and 8 out of 12 had confirmed heterozygous loss-of-function mutations within the coding region of the PAX6 gene. Using an ICA and dual-regression approach, common ICNs were identified in all participants and groups were contrasted in whole-brain activity correlated with these networks. The three ICNs of interest were the executive control network, the primary visual network, and the default mode network. In all network comparisons the aniridia group showed regions of increased functional connectivity relative to controls; there were no regions in which the control subjects had stronger functional connectivity.

In the frontal-cortex-based executive control network, the aniridia group showed greater connectivity than control subjects in a number of occipital cortical regions. The recruitment of posterior visual regions to this anterior network may represent a need to supplement insufficient frontal function, as observed in the only previous fMRI study on individuals with aniridia (Ellison-Wright et al., 2004), or a greater degree of involvement of executive control in visual processing. Prefrontal cortex is involved in top-down modulation of visual processing based on current goals (Miller and Cohen, 2001; Gazzaley et al., 2007), and the increased connectivity between these regions in the aniridia group could be related to an increased demand for executive control due to altered visual input.

A similar pattern was observed in the analysis of the primary visual network: participants in the aniridia group showed greater intrinsic connectivity than controls in left fusiform gyrus, a region which is not typically part of this network in healthy individuals. This suggests that individuals with aniridia are utilizing proximal cortical regions to supplement the functioning of the hub of this network, possibly due to a lack of efficiency within the network proper. This inefficiency may be caused directly by structural abnormalities related to altered PAX6 gene function (Free et al., 2003; Ellison-Wright et al., 2004) or by the ocular deficits associated with aniridia (e.g., poor visual acuity, cataract, glaucoma, foveal hypoplasia) which necessarily reduce the efficacy of functional input to the occipital lobe.

Finally, in the default mode network, some of the regions (medial temporal and frontal) that showed increased functional connectivity were similar to those reported by Ellison-Wright et al. (2004) to have altered structure or task activation in a single family with aniridia. Hippocampal and medial frontal regions have been noted in some studies to be part of the normal default mode network (Vincent et al., 2006; Buckner et al., 2008), and the increased connectivity in nearby parahippocampal and inferior and lateral frontal cortex in the current study suggests that the functioning within the default mode network in aniridia was reduced compared to controls such that these adjacent regions were recruited.

When considering the functional connectivity across all three ICNs, it is evident that the regions where the aniridia group exhibited greater connectivity than controls were primarily located within occipital and frontal cortex, as well as the medial temporal lobe. The occipital lobe constitutes the core of visual processing in the brain, which is the sensory modality most notably disrupted by heterozygous loss-of-function mutations of the PAX6 gene. Furthermore, reductions in gray matter concentration in the occipital lobe have been identified previously in individuals with aniridia (Free et al., 2003). White matter deficits in occipital cortex and gray matter volume excesses in frontal and medial temporal lobes also have been reported in individuals with aniridia, in addition to reduced task-based activation of frontal regions during a verbal executive function paradigm (Ellison-Wright et al., 2004). These differences suggest that PAX6 plays a particularly active role in the development of these regions and structural abnormalities may lead to these regions being recruited to a greater extent during task-free activity in order to bolster the efficiency of the network, potentially allowing for better functioning during active behaviors (Sharp et al., 2011).

Individuals with PAX6-mediated aniridia have insufficient levels of PAX6 protein, which is expressed in the eye, cerebellum, olfactory bulb and throughout the cerebral cortex in early human brain development, but is not expressed cortically in the adult (Walther and Gruss, 1991; Stoykova and Gruss, 1994; Georgala et al., 2011). The differences in functional connectivity demonstrated in this study likely represent complex and compounding effects of PAX6 haploinsufficiency. These aberrant patterns of activity in the adult could be a product of: (1) inefficient local neural circuits, as a direct consequence of functional PAX6 reduction or through putative genomic targets involved in connectivity during early cortical development; (2) irregular connectivity due to the underdevelopment of visual and olfactory sensory input structures; (3) cortical reorganization in late-stage brain development due to a lack of or atypical environmental sensory input; or (4) combined effects of the above. The results of the current study reflect differences in large-scale functional network dynamics; given that PAX6 has an extensive role in early neural development and has noteworthy anatomical and functional effects on sensory structures, the findings of the current study are ostensibly due to an amalgam of direct molecular effects of PAX6 in early development and indirect consequences of these effects occurring later in the lifespan once PAX6 is no longer expressed.

In regards to the present findings, there are a few potential sources of error that should be considered. First, structural abnormalities were not measured in the current sample and, thus, conclusions about the relationship between structure and function were based on previously reported structural differences in several brain regions in aniridia (Free et al., 2003; Ellison-Wright et al., 2004). As such, one cannot exclude the possibility that disrupted local gray matter structure or altered structural connectivity in the current aniridia sample contributed to the observed differences in functional connectivity in an undetermined manner. Additionally, in all of the group ICN comparisons, the aniridia group exhibited regions of increased connectivity whereas the control group exhibited no such regions. This leads to the question of whether increased connectivity or diffuse functional networks are a non-specific feature of intrinsic functional neural organization in aniridia. Further research is required to investigate this possibility in other individuals with aniridia using alternative analytical approaches. It also may be fruitful to explore the developmental trajectory of these connectivity patterns in aniridia to clarify the interaction of functional experience with structural changes in formation of these networks.

## Conclusions

This study investigated intrinsic functional connectivity in individuals with aniridia compared to healthy controls. Increased connectivity was observed in the aniridia group in three ICNs: the executive control, primary visual, and default mode networks. Specifically, individuals with aniridia recruited supplementary occipital, frontal, and medial temporal regions not identified in matched controls, suggesting a lack of network specificity and refinement related to mutations of the PAX6 gene. Though the specific causes of these functional connectivity differences remain undetermined at present, mutations in the PAX6 gene evidently do interfere with the formation of discrete cognitive networks and necessitate greater functional involvement of additional neural regions. Further research is required to investigate how these unique patterns of functional co-activations in aniridia derive from neurophysiological mechanisms of PAX6 at the cellular and molecular level, as well as how they may contribute to behavioral differences.

## Conflict of interest statement

The authors declare that the research was conducted in the absence of any commercial or financial relationships that could be construed as a potential conflict of interest.
